# Research platform for fibromyalgia in Japan

**DOI:** 10.1186/ar3560

**Published:** 2012-02-09

**Authors:** Kusuki Nishioka

**Affiliations:** 1 Japan College of Fibromyalgia Investigation, Japan Rheumatism Foundation, Institute of Medical Science, Tokyo Medical University, Tokyo, Japan

## Epidemiology

Fibromyalgia (FM) is found worldwide, with an estimated prevalence of 1% to 4% of the general population. In Japan epidemiological surveillance showed number of the patient up to1.66% in population-based studies in 2003, following 2.04% by internet surveillance in 2011. 3,500 Japanese patients with our FM data base, frequent age of onset is 35 to 55 years, estimated prevalence of FM, reaching 40% in woman of age 30 to 50 years old. FM occurs in children and adolescents, although only a few epidemiologic studies.

## Pathogenesis

FM was initially one the kind for inflammatory muscle pain. Following this concept FM was classified so called soft-tissue rheumatism. Now FM is recognized CNS sensitization following functional pain caused by neuroendocrine system and stress. The abundant neuroendocrine and pain regulation descending pathway and sensitization of pain receptor target molecules such as α_2_δ ligand, LPA and collapsing response mediator, where currently protein (CRMP) family have been revealed.

## New diagnosis, provisional ACR 2010 criteria

1990, ACR proposed FM criteria based on 18 tender point sites on digital palpation with exclusive differential diagnosis. In 2010, provisional criteria is score based on total score of severity for pain and somatic symptom (2010 ACR criteria). Based on 2010 ACR criteria, we proposed the assessment of FM severity termed "FAS31". Here, we would like propose the assessment of FM severity score termed FAS31.

FDA approved of pregabalin in FM by double-blind, multicenter and randomized study. Both studies enrolled patients with a diagnosis of FM using the ACR criteria. Each of these studies showed a significant reduction in pain compared with placebo. In addition, improvement demonstrated based on FIQ. In Japan, this clinical trial has been developed. Sooner or later, excellent result will be revealed.

In other medication, gabapentin practical efficacy for reduced pain with FM patient. Several anti-dispersants (SSTIs, SNRIs. TCAs) NSAIDs, muscle relaxant, anti epileptics and pilocarpine hydrochloride also reduced the pain and an associated symptom. Based on with multivariant statistical analysis based on 3,500 patients, we will present several associated somatic symptoms influencing on drug response for pain and prognosis with FM.

In conclusion, FM is one the most important scientific field to understand the pain neurology and rheumatology in near.

